# Relevance of ^18^F-DOPA visual and semi-quantitative PET metrics for the diagnostic of Parkinson disease in clinical practice: a machine learning-based inference study

**DOI:** 10.1186/s13550-023-00962-x

**Published:** 2023-02-13

**Authors:** Alex Iep, Mohammad B. Chawki, Lucas Goldfarb, Loc Nguyen, Vincent Brulon, Claude Comtat, Vincent Lebon, Florent L. Besson

**Affiliations:** 1 Nuclear Medicine Department, Service Hospitalier Frédéric Joliot SHFJ-CEA, Orsay, France; 2 Inserm, CNRS, CEA, Laboratoire d’Imagerie Biomédicale Multimodale BioMaps, SHFJ, Université Paris Saclay, Orsay, France

**Keywords:** Fluorodopa F 18, Parkinson's disease, Machine learning, Positron-emission tomography

## Abstract

**Purpose:**

To decipher the relevance of visual and semi-quantitative 6-fluoro-(18F)-L-DOPA (^18^F-DOPA) interpretation methods for the diagnostic of idiopathic Parkinson disease (IPD) in hybrid positron emission tomography (PET) and magnetic resonance imaging.

**Material and methods:**

A total of 110 consecutive patients (48 IPD and 62 controls) with 11 months of median clinical follow-up (reference standard) were included. A composite visual assessment from five independent nuclear imaging readers, together with striatal standard uptake value (SUV) to occipital SUV ratio, striatal gradients and putamen asymmetry-based semi-quantitative PET metrics automatically extracted used to train machine learning models to classify IPD versus controls. Using a ratio of 70/30 for training and testing sets, respectively, five classification models—k-NN, LogRegression, support vector machine, random forest and gradient boosting—were trained by using 100 times repeated nested cross-validation procedures. From the best model on average, the contribution of PET parameters was deciphered using the Shapley additive explanations method (SHAP). Cross-validated receiver operating characteristic curves (cv-ROC) of the most contributive PET parameters were finally estimated and compared.

**Results:**

The best machine learning model (k-NN) provided final cv-ROC of 0.81. According to SHAP analyses, visual PET metric was the most important contributor to the model overall performance, followed by the minimum between left and right striatal to occipital SUV ratio. The 10-time cv-ROC curves of visual, min SUVr or both showed quite similar performance (mean area under the ROC of 0.81, 0.81 and 0.79, respectively, for visual, min SUVr or both).

**Conclusion:**

Visual expert analysis remains the most relevant parameter to predict IPD diagnosis at 11 months of median clinical follow-up in ^18^F-FDOPA. The min SUV ratio appears interesting in the perspective of simple semi-automated diagnostic workflows.

## Introduction

Idiopathic Parkinson disease (IPD) is the second neurodegenerative disorder worldwide, progressively affecting the deep brain dopaminergic pathways [1]. Because dopaminergic loss starts years before symptoms, the related clinical hallmarks—shaking, stiffness, and difficulty with coordination—may be subtle at very early stages, delaying definitive clinical diagnosis [2–4]. Although clinical and research diagnostic criteria for Parkinson disease have been updated in 2015 [5–7], the diagnostic confidence of IPD is still massively based on clinical follow-up and response to levodopa therapy, the new movement disorder society clinical diagnostic criteria for IPD being judged not useful by the experts in real-life practice [5]. Nuclear imaging has been shown a very useful complementary diagnostic tool [8], especially in atypical cases or at early stages of the disease. Dopaminergic PET imaging has gained progressive interest in practice, due to higher spatial resolution, faster acquisition procedure, improved dosimetry and cost-effectiveness compared to dopamine transporter (DAT) single photon emission computed tomography (SPECT) [9, 10]. As for DAT SPECT, visual assessment remains the most widely used interpretation method in practice [11]. Although simple static PET semi-quantification showed similar performance compared to PET kinetic procedures [12, 13], its diagnostic performance compared to visual assessment remains largely unexplored in ^18^F-DOPA PET imaging of IPD.

Taking advantage of emerging machine learning capabilities, the aim of this study was to robustly decipher the relevance of visual and semi-quantitative ^18^F-DOPA interpretation methods for the diagnostic of IPD in PET/MRI.

## Material and methods

### Population

**Fig. 1:**
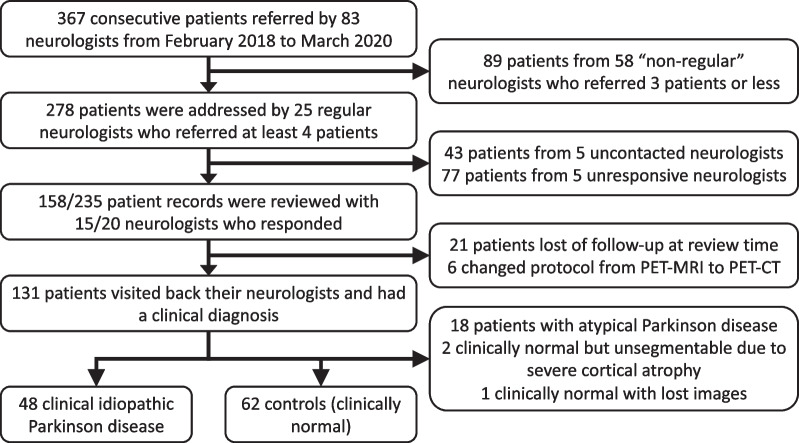
Flowchart of the patient selection process. From 367 patients, 110 were finally retained, including 48 IPD and 62 controls

In this monocentric controlled study, all consecutive patients addressed in our PET/MR imaging center for suspicion of Parkinsonism were screened from February 2018 to March 2020. From the initial database, 367 patients from 83 different doctors were retrieved. Fifteen neurologists out of the 25 ones who addressed at least four patients responded back. All the included patients fulfilled the following inclusion criteria: The clinical diagnosis was based on medical history, clinical symptoms (bradykinesia, rigidity, tremor), symptoms evolution under dopaminergic agonist; 11 months of median clinical follow-up between ^18^F-DOPA PET and the last consultation date was verified. Based on neurologists’ expertise, the clinical follow-up separated our population into two groups: clinically confirmed Parkinsonian syndrome and non-Parkinsonian syndromes (essential tremor, neuroleptic side-effects, non-Parkinsonian gait disorders). The cases of Parkinsonism still judged atypical after full multidisciplinary diagnostic work-up (in-laboratory video polysomnography, orthostatic hypotension test, specialized consultation in memory, neuro-psychologic test, assessment for dysarthria or swallowing trouble) were excluded from our study. A general overview of the selection process is provided in Fig. 1.

### ^18^F-DOPA PET/MRI protocol

All the included patients underwent hybrid brain imaging on the same 3-T PET/MRI system (Signa PET/MRI, GE Healthcare). The imaging protocol fulfilled the international guidelines [11], and all patients discontinued L-DOPA at least 12 h as recommended by manufacturers. A simultaneous single-bed PET/MRI acquisition of 10 min performed 1h30 after the intravenous injection of 1.5 MBq/kg of ^18^F-DOPA. During the PET acquisition, standard brain MR pulse sequences were performed, in particular: a zero time echo (ZTE) pulse sequence for MRI-based attenuation correction (matrix size: 128 × 128; flip angle: 5°; TE: 1.7 ms; TR: 4 ms; slice thickness: 2.78 mm) and a morphological 3D T1-weighted pulse sequence, BRAVO, matrix size: 256 × 226; flip angle: 15°; TE: 3.2 ms; TR: 8.5 ms; slice thickness: 1.2 mm). All the PET data were corrected from attenuation and reconstructed using an iterative algorithm, 3D TOF-OSEM, 8 iterations and 28 subsets with time of flight and point spread function modeling: matrix size 256 × 256 × 89, voxel size, 1.2 × 1.2 × 2.8 mm with a 3-mm Gaussian post-filtering.

### Image processing, data extraction and preparation

**Fig. 2:**
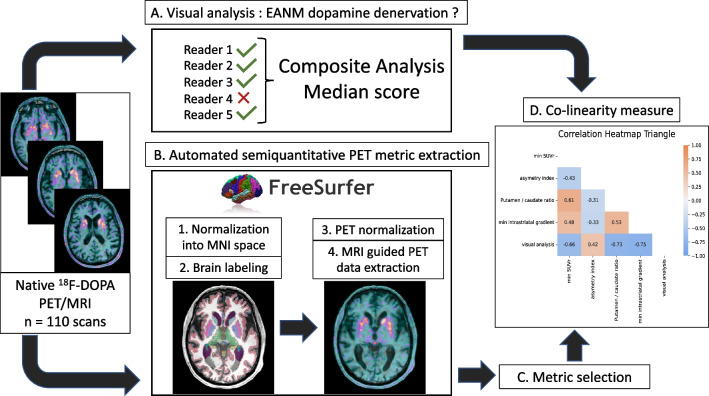
Image processing and data extraction. The whole dataset was assessed both visually and automatically. For visual analysis (A), five independent readers blindly re assessed all the ^18^F-DOPA PET data by using the international recommendation procedure guideline. An overall composite “visual” interpretation based on the five readers results was generated for each ^18^F-DOPA PET. For automated analysis (B), the ^18^F-DOPA PET/MRI data were processed in a dedicated neuroimaging pipeline (Free surfer) to be standardized and labeled. For this purpose, each T1w MRI was normalized into the MNI space, and the transformation was applied to the corresponding ^18^F-DOPA PET. Numerous metrics could be extracted automatically from the standardized PET data. Finally, a subset of targeted PET metrics was retained (C) based on their conceptual relevance and explored to identify potential high collinearity (D)

An overview of image processing and analysis is provided in Fig. 2.

For the PET visual analysis, five experts in nuclear hybrid imaging, whose experience ranged from 6 to 12 years, independently reviewed all the PET/MRI on the same imaging workstation (ADW, version 4.7), all blinded to the final clinical diagnosis. For this purpose and using the predefined international criteria for ^18^F-DOPA PET imaging [11] with the same color scale, each expert realigned the data along the anterior to posterior corpus callosum axis, scaled the PET images to the rostral part of the putamen and carefully analyzed the distribution and intensity of ^18^F-DOPA uptake in the striatum (caudate and putamen) and background. For each case, the procedure was timed from the image opening to the end of the analysis process. Any asymmetry or decrease in uptake in the striate was considered pathological. A three-point scale normal (“− 1”), undetermined (“0”), and pathological (“1”) was first used, and the median score of the five readers represented the intermediate composite analysis of their expertise. In another session, readers altogether reviewed undetermined median score (“0”) cases to reach a consensus, and results were consequently attributed to all five readers to obtain a final composite binary output: normal or pathological. The final composite score was compared to the clinical standard reference for true positive (TP), false positive (FP), false negative (FN) and true negative (TN) rates.

For the semi-quantitative analyses, several PET metrics were automatically extracted by using FreeSurfer (v7.1.1), an open-source neuroimaging toolkit which provides advanced automated full processing streams for MRI and PET standardization and data extraction. T1-weighted MRI of all the patients was normalized to the Montreal neurological institute (MNI) reference space by using a complete neuro-imaging automated pipeline including nonlinear registration [14–16], intensity correction [17] and skull stripping [18]. The cortical and subcortical brain structures were automatically segmented and labeled [19]. The Desikan–Killiany–Tourville segmentation mask was applied to the corresponding PET data without partial correction volume. From the segmented ^18^F-DOPA PET/MRI, SUVmean were automatically extracted by using the Freesurfer’s PET/MRI module named Petsurfer [20, 21]. Because the striatum contralateral to the most affected clinical side typically reflects the most altered SUV ratio in idiopathic Parkinson disease, the minimum SUVmean ratio of Freesurfer-based metrics showing between-group statistical significance on first-line descriptive analyses was retained because of their well-grounded physiopathological relevance: the minimum between $\frac{{\text{Right striatum}}}{{\text{Occipital cortex}}}$ and $\frac{{\text{Left striatum}}}{{\text{Occipital cortex}}}$ SUV ratio (min SUVr) [22–24], the minimum between $\frac{{\text{Right putamen}}}{{\text{Right caudate nucleus}}}$ and $\frac{{\text{Left putamen}}}{{\text{Left caudate nucleus}}}$ SUV ratio (P/C gradient) [25], the minimum between $\frac{{\text{Right posteriot putamen}}}{{\text{Right anterior putamen}}}$ and $\frac{{\text{Left posteriot putamen}}}{{\text{Left anterior putamen}}}$ SUV ratio (intra-striatal gradient) [26] and asymmetry index [27] for Putamen SUVmean uptake, computed as follows: $\frac{|{\text{Left Putamen-Right Putamen}}|}{{\text{Left Putamen+Right Putamen}}}$. Finally, potential collinearity between the five ^18^F-DOPA PET metrics (binary visual score and four semi-quantitative) was searched before machine learning procedures by using Spearman nonparametric measure of rank correlations.

### Deciphering the contribution of ^18^F-DOPA PET parameters for the identification of IPD

**Fig. 3:**
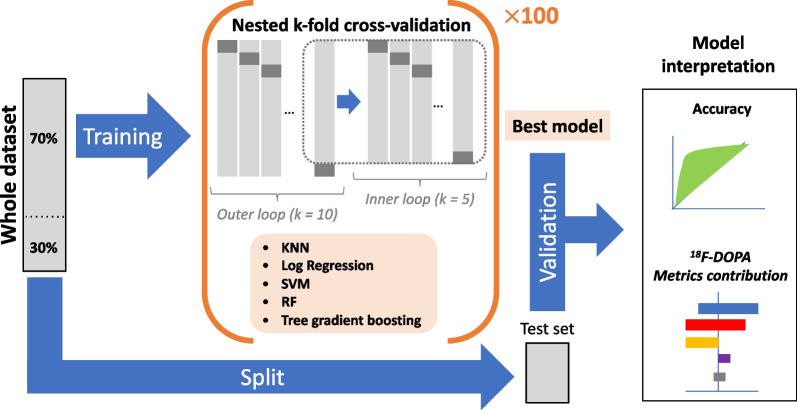
Statistical analyses. After identifying potential collinearity between the PET metrics, the whole dataset (visual binary interpretation and semi-quantitative PET metrics from 110 ^18^F-DOPA PET/MRI) was split into training (70%) and test (30%) sets. Five machine learning classifiers (KNN, Log Regression-Log Reg, Support Vector Machine-SVM, Random Forest-RF and tree gradient boosting) were trained to predict the final diagnosis at 11 months of median follow-up (IPD or control) on the training set by using a nested k-fold cross-validation procedure. (Each model parameters are fine-tuned and cross-validated while optimizing the bias of over fitting.) The overall nested cross-validation procedure was repeated 100 times. The best model on average was applied on the test set to provide general unbiased accuracy. Finally, the contribution of each ^18^F-DOPA PET parameter (the visual and four semi-quantitative metrics) in the model predictions was deciphered

The general framework of the statistical analyses is provided in Fig. 3. Considering the ultimate diagnosis retained by the neurologists at 11 months of median follow-up, five different embedded machine learning classifiers—k-nearest neighbors (k-NN), Log Regression with L1 and L2 regularization (Elastic Net, Log Reg), Support vector Machine (SVM), Random Forest (RF) and tree gradient boosting (XGBoost)—were learned patient-wise and compared on their capability to predict the final diagnosis of the patients based on the five selected ^18^F-DOPA PET metrics: binary visual score, min SUVr, P/C gradient, intra-striatal gradient and asymmetry index. For this purpose, the dataset was split into training and test sets with a 70%/30% ratio. To prevent overfitting and improve the robustness of the 5 models’ performance, a nested k-fold cross-validation was used during the training phase [28, 29]. For each model, the hyperparameters tuning was nested under the model selection (inner loop, *k* = 5), model which was assessed on random subsamples of the training set (outer loop, *k* = 10). The overall nested scheme was repeated 100 times. At the end, the best predictive model in average was applied on the testing set to provide unbiased optimized accuracy to predict the diagnosis of IPD. The explainability of the best model was assessed by using the Shapley additive explanations method (SHAP) (https://christophm.github.io/interpretable-ml-book/shap.html), which deciphers the contribution of each ^18^F-DOPA PET parameter. Finally, cross-validated ROC curves of the best model performance (*k* = 10) with the most relevant PET parameters were generated and compared. All the statistical analyses were performed with Python (version 3.8; Python Software Foundation) [30], using pandas, numpy, scipy, scikit learn and xgboost libraries.

## Results

### Population study

From the initial 367 patients addressed in our PET/MRI unit for suspicion of Parkinsonism, a total of 158 medical record has been reviewed. After accounting for excluded patients—lost in follow-up at review time (*n* = 21), who changed protocol because of MRI contraindications (*n* = 6), with atypical Parkinsonism (*n* = 18) and with unsegmentable brain due to severe atrophy in Freesurfer automatic process (*n* = 1) with lost data (*n* = 1)—a total of 110 patients were included. Finally, 110 patients were retained for the analyses, including 48 IPD and 62 patients considered clinically normal (control group). As illustrated in Table 1, no between-group statistical significance was observed for age, sex, nor clinical follow-up. Patients median age was 72 and 76 years old in IPD and control groups, respectively (*p* = 0.126). The IPD group sex ratio was imbalanced compared to the control group, but without statistical significance (M/F = 29/19 vs. 32/30; *p* = 0.360). The overall median clinical follow-up was 330 days, with longer duration for the IPD group compared to the control group (570 vs. 296 days, *p* = 0.054).

**Table 1:** Patients’ characteristics

	Idiopathic Parkinson disease (IPD)	Control group	*p* value
Number of patients	48	62	–
Sex ratio M/F	29/19	32/30	0.360
Age (years)			
Median	72	76	0.126
[IQR]	[67–79]	[69–82]	
[min–max]	[39–92]	[45–93]	
Clinical follow duration (days)			
Median	570	296	0.054
[IQR]	[215–730]	[116–501]	
[min–max]	[23–992]	[29–927]	

*IQR* interquartile range

### Image processing, data extraction and preparation

**Fig. 4:**
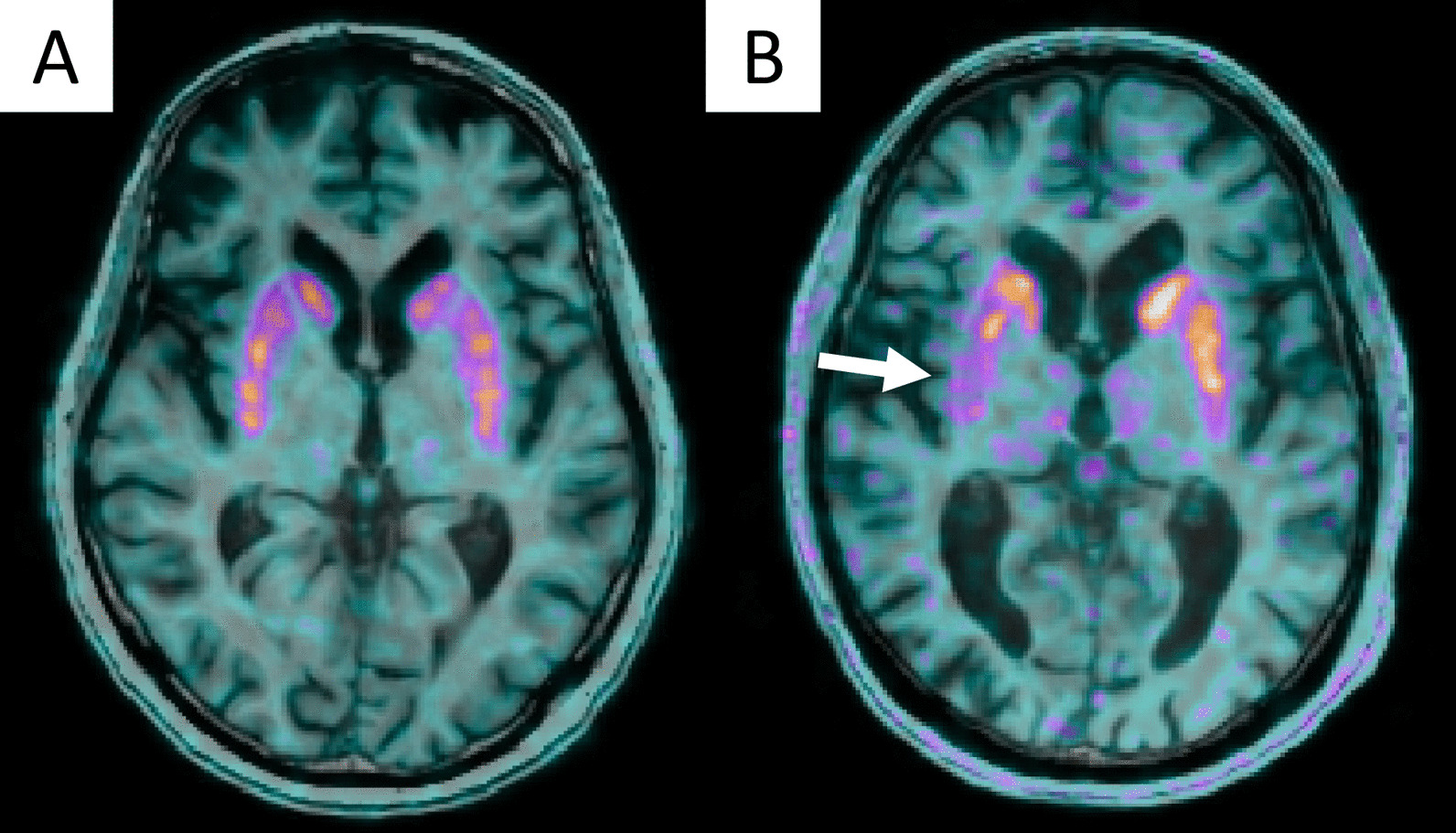
Visual analyses of ^18^F-DOPA PET/MRI according to the international guidelines [11]. **A** This IPD patient, visually considered normal. The clinical follow-up and had symptoms relief after L-DOPA therapeutic test classified him as FN. Only min SUVr was below the 95% confidence interval estimated from controls. **B** This IPD presented unilateral right posterior putamen (white arrow) pre-synaptic dopamine denervation and global striatal ^18^F-DOPA decrease with faintly increased background unspecific activity. PET right putamen dopamine denervation was confirmed with symptoms relief after L-DOPA therapeutic test

**Fig. 5:**
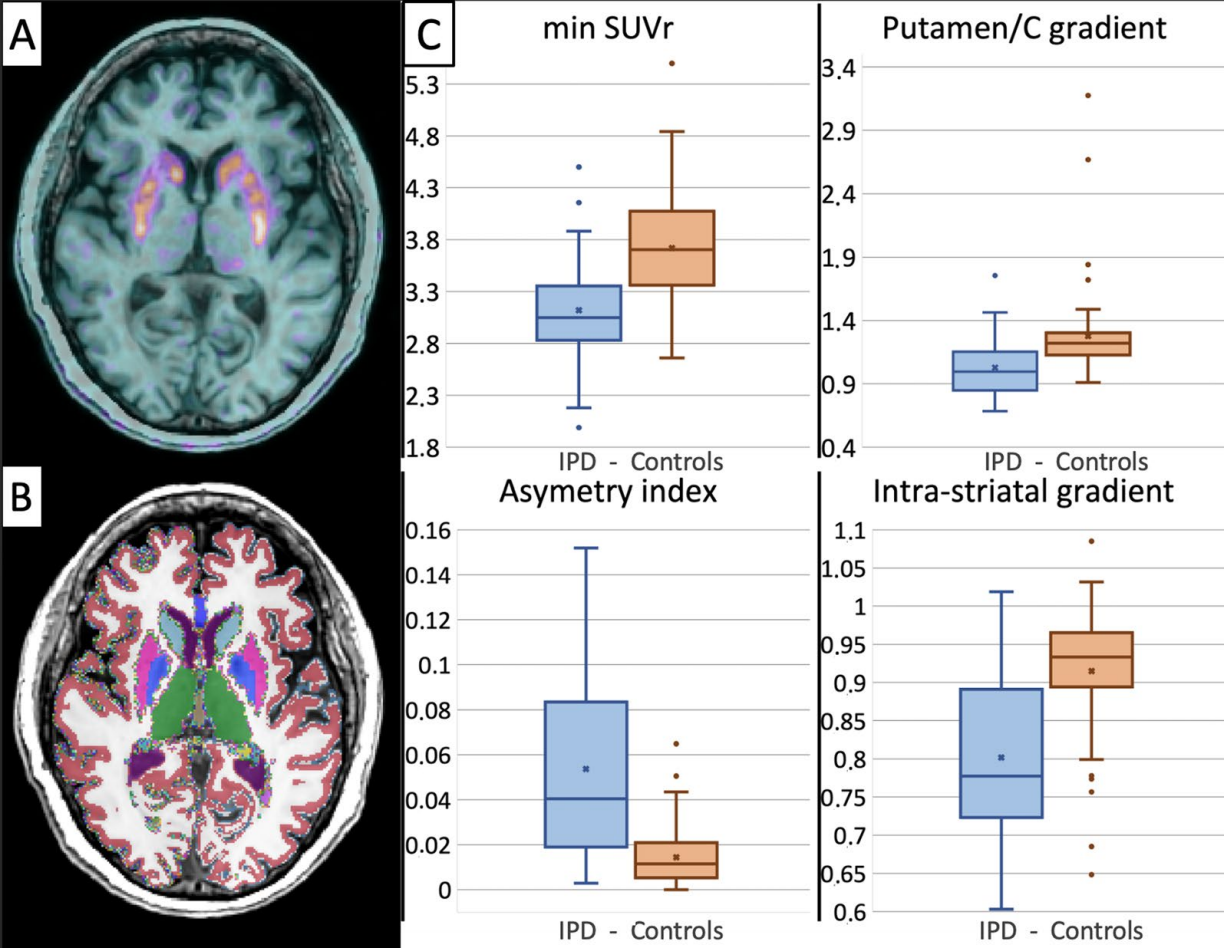
Subgroups distributions of ^18^F-DOPA PET semi-quantitative metrics. **A** fused ^18^F-DOPA PET/T1weighted MRI normalized in the MNI space of a control subject. **B** MNI-normalized T1-weighted MRI, overlaid with corresponding Freesurfer-based automated segmentation labels (**C**). Subgroups distribution of the four ^18^F-DOPA PET extracted metrics, for which a statistical between group difference was verified, values and *p* values are detailed in Table 3

**Fig. 6:**
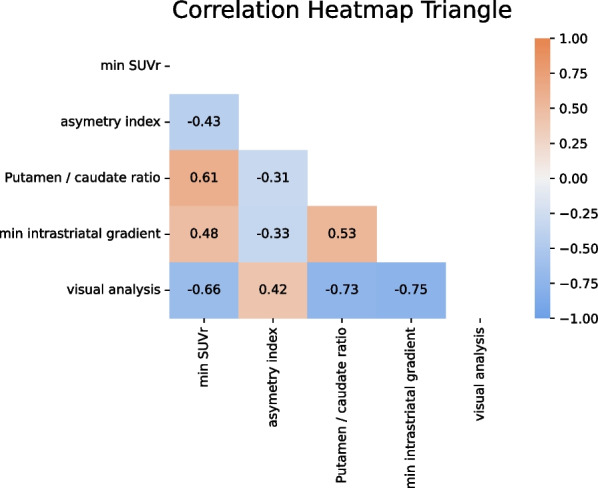
Heatmap of the between PETmetrics correlations (Spearman rank test)

Results of readers final composite score are reported in Table 2. Considering a visual interpretation time of less than 2 min on average, the final composite score of the five readers provided 32/110 TP, 4/110 FP, 16/110 FN, and 58/110 TN, resulting in overall sensitivity, specificity, positive predictive value, and negative predictive value of 66,7%, 93.5%, 88.9%, and 78.4%, respectively. Two illustrative cases of visual analysis are provided in Fig. 4. The distribution of the four ^18^F-DOPA PET semi-quantitative metrics is illustrated in Fig. 5 and provided in Table 3. As predefined in the selection process, the between-groups differences were significant for the four semi-quantitative PET metrics. The inter-correlations between the five PET metrics (visual and four semi-quantitative PET metrics) ranged from − 0.75 (pair visual and intra-striatal gradient) to 0.61 (pair min SUVr and P/C gradient) (Fig. 6), all of them being statistically significant (Spearman rank test, *p* < 0.05).

**Table 2:** Readers visual analyses, majority vote results, and mean interpretation duration

	Diagnostic performances		Mean interpretation duration (min) [95%CI]
Reader 1	TP = 30.0% (33/110)	FP = 3.6% (4/110)	1.09 [1.06–1.11]
	FN = 13.6% (15/110)	TN = 52.7% (58/110)	
Reader 2	TP = 29.1% (32/110)	FP = 3.6% (4/110)	0.99 [0.95–1.03]
	FN = 14.5% (16/110)	TN = 52.7% (58/110)	
Reader 3	TP = 28.2% (31/110)	FP = 3.6% (4/110)	1.44 [0.95–1.03]
	FN = 15.5% (17/110)	TN = 52.7% (58/110)	
Reader 4	TP = 28.2% (31/110)	FP = 10.9% (12/110)	1.97 [1.92–2.01]
	FN = 15.5% (17/110)	TN = 45.5% (50/110)	
Reader 5	TP = 20.0% (22/110)	FP = 2.7% (3/110)	0.7 [0.65–0.75]
	FN = 23.6% (26/110)	TN = 53.6% (59/110)	
Majority vote	TP = 29.1% (32/110)	FP = 3.6% (4/110)	1.24 [1.20–1.28]*
	FN = 14.5% (16/110)	TN = 52.7% (58/110)	

Mean interpretation time was expressed with 95% confidence interval [95%CI]

*TP* true positive, *FP* false positive, *FN* false negative, *TN* true negative

*Pooled mean interpretation duration across the five readers

**Table 3:** Characteristics of the semi-quantitative metrics extracted from normalized ^18^F-DOPA PET data

	IPD	Controls	*p* value
Min SUVr median [IQR]	3.04 [2.81–3.34]	3.70 [3.37–4.07]	1.60 × 10^−7^
Asymmetry index median [IQR]	0.041 [0.019–0.083]	0.011 [0.005–0.021]	2.17 × 10^−8^
Putamen/caudate gradient median [IQR]	1.00 [0.85–1.15]	1.22 [1.13–1.30]	1.76 × 10^−7^
Min intra-striatal gradient median [IQR]	0.78 [0.73–0.89]	0.93 [0.89–0.96]	1.31 × 10^−7^

All ratios are unitless and expressed as median with interquartile range [IQR]

### Deciphered contribution of ^18^F-DOPA PET parameters for the identification of IPD

**Fig. 7:**
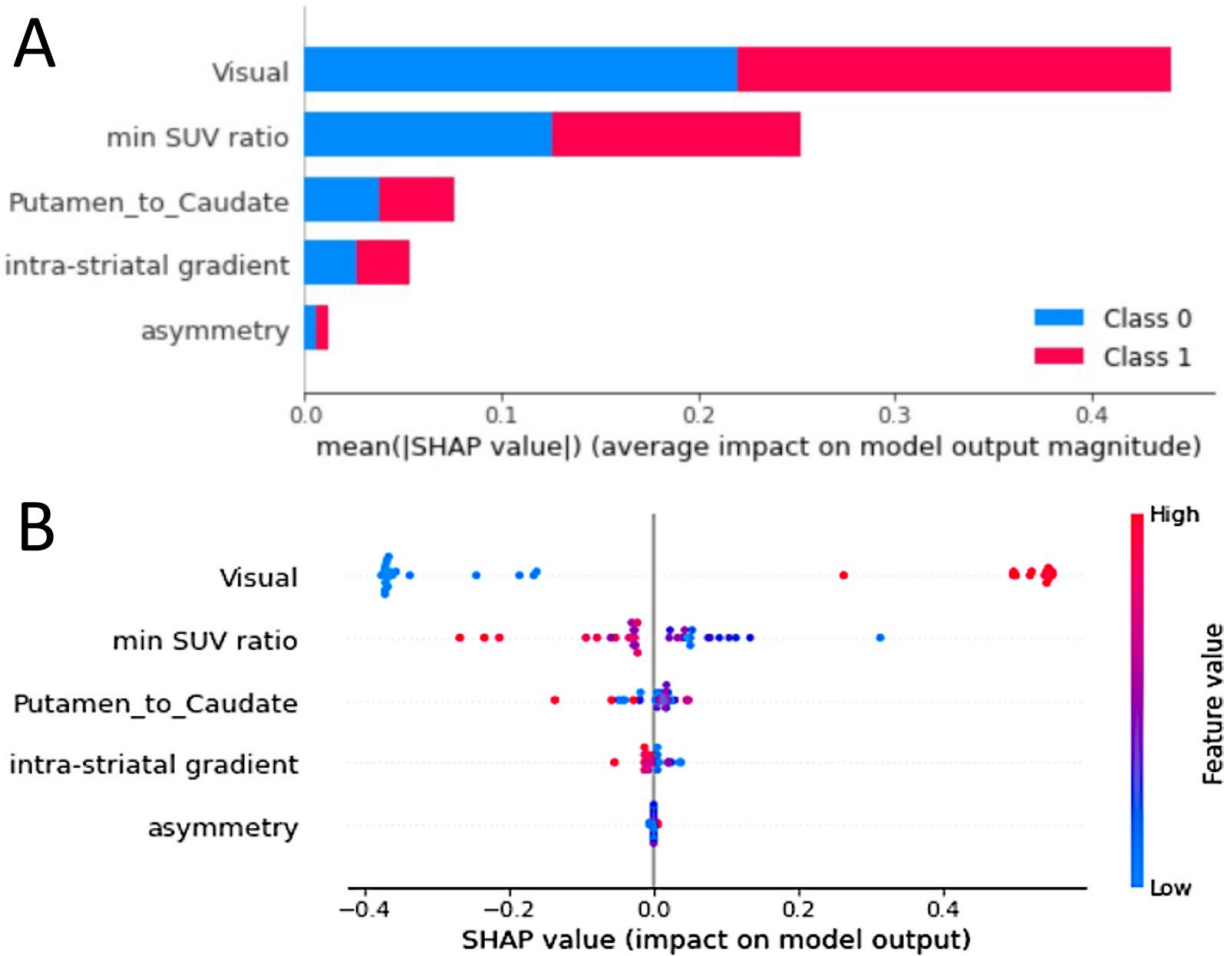
Shap plot of features’ contribution on the best classifier model output. **A** Stacked bars of absolute value of the SHAP values for each feature sorted by their importance across all patients for classification. “Class 0” corresponds to the controls and “Class 1” to the IPD group. For classification of IPD versus control, PET visual score is the most contributive parameter, then min SUV ratio is the second most contributive parameter. The three others do not appear relevant for the classification of IPD versus controls here. **B** SHAP distribution on *x*-axis of the five features for every patient and control. The impact is correlate to the absolute value of the SHAP value. The higher (redder) feature value, the more it contributes to IPD classification. On the opposite the lower (bluer) feature value, the more it contributes to control classification. Min SUV ratio and intra-striatal gradient are inversely correlated to IPD classification as represent dopamine denervation at different levels

**Fig. 8:**
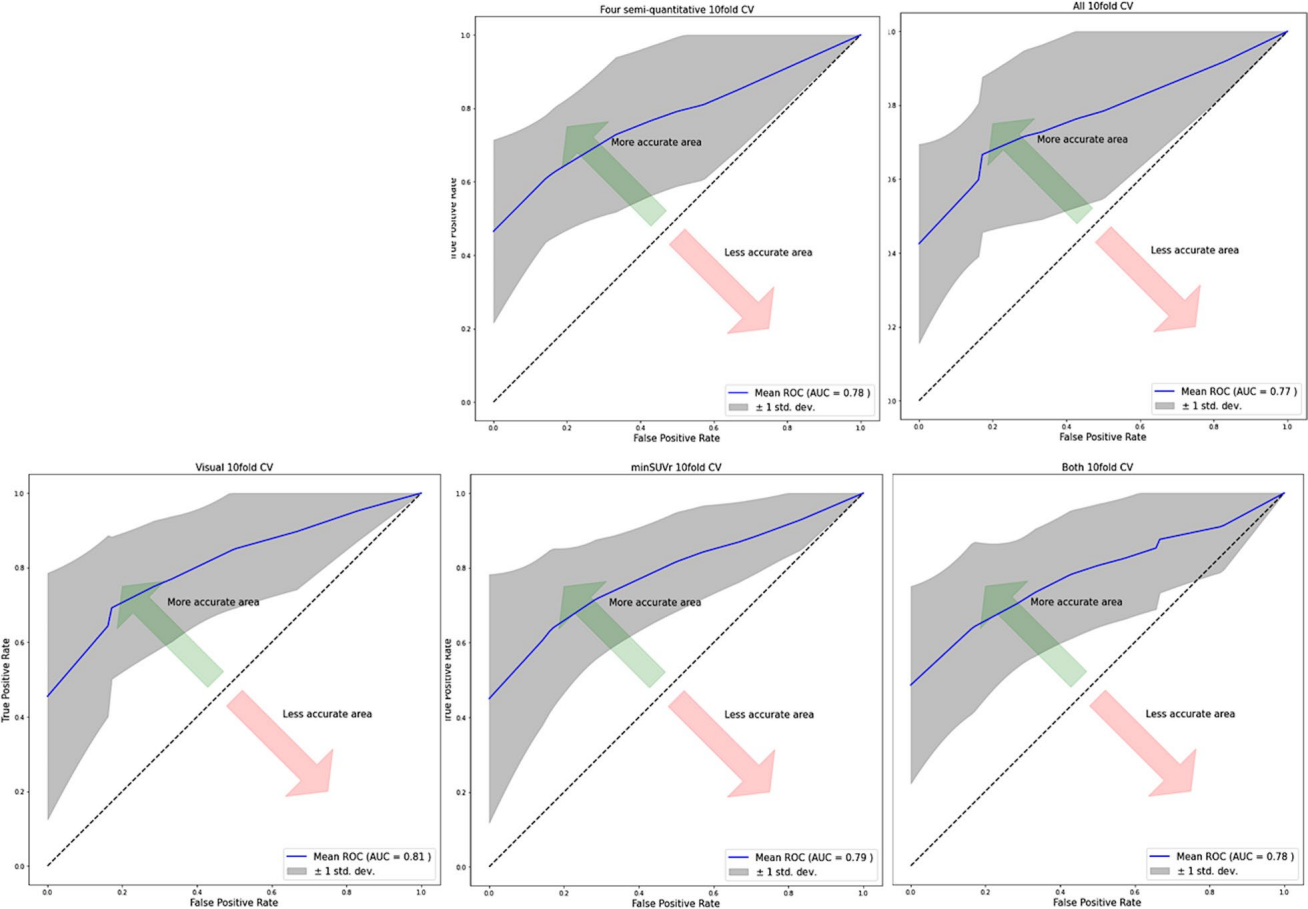
ROC curves comparison. Left panel: best model AUC (tenfold cross-validated) with visual PET metric alone; middle panel: best model AUC (tenfold cross-validated) with min SUVr PET metric alone; and right panel: best model AUC (tenfold cross validated) with both visual and min SUVr PET metrics. The visual mean AUC of visual and min SUVr were similar (0.81). Combining the two parameters did not improve the overall accuracy (0.79)

None of the four PET metrics being highly correlated (Spearman $\left|\rho \right|$ < 0.9), all were included as input in the machine learning procedures. As provided in Table 4, all the classifiers provided overall good performance to classify IPD and controls. The best classifier was on average the k-NN scheme. This model with fine-tuned hyperparameters provided final test area under the curve (AUC) of the cv-ROC of 0.81. SHAP analyses showed that visual PET metric was the most important contributor to the model overall performance, followed by the min SUVr (Fig. 7). Finally, the cross-validated ROC curves of visual, min SUVr or visual combined to min SUVr showed similar performance between visual or semi quantitative metrics (Fig. 8).

**Table 4:** Models’ performance on average, after 100 nested cross-validation procedures

Model	Mean accuracy (std)
k-NN	80.9 ± 1.55
Log regression (Elastic Net)	80 ± 1.44
Random forest	78.1 ± 2.25
SVM	78.4 ± 1.46
XGBoost	79.3 ± 2.5

After 100 iterations, the k-NN scheme was the best model on average

## Discussion

In our controlled study including 110 participants with a median clinical follow-up reaching 11 months, the performance of five machine learning procedures with 100-time nested cross-validation were compared to accurately classify IPD from control. Our results demonstrated that among different well-grounded standard PET metrics, visual assessment and semi-quantitative min SUV ratio provided similar performance. In DAT SPECT, despite improvements with CZT systems, the spatial resolution and sensitivity detection remains lower than PET-CT systems, together with the lack of anatomical repairs for accurate realignment of the brain motivated the used of semi-quantitative ratios to limit inter reader variabilities. Although semi-quantification showed increased diagnostic performance in few SPECT studies [31–33], such approach does not preclude from interobserver variations if performed manually or semi-automatically, and is still considered as an adjunct to visual analysis. Although aromatic l-amino acid decarboxylase (AADC) striatal deficiency can be quantified by PET dynamic acquisitions in Parkinsonian syndromes [34], practical consideration and similar diagnostic performance in cross-sectional studies have led to promote simpler SUV ratios. DAT SPECT and ^18^F-DOPA PET visual interpretation share similar features but have two different targets in dopaminergic neurotransmission that can decrease in parallel but not necessarily synchronously with progression of neurodegenerative Parkinsonism. In theory, in early IPD compensatory mechanisms trigger presynaptic DATs expression is downregulation, and l-amino acid decarboxylase upregulation which is was confirmed in a meta-analysis [22, 23, 25, 35] demonstrating that AADC defect seen with ^18^F-DOPA PET is consistently smaller than DAT defects in SPECT studies. Nevertheless both are able to diagnose presynaptic dopaminergic deficits in early phases of PD with excellent sensitivity and specificity [11], to our knowledge no study showed superiority of any procedure and compared to SPECT, ^18^F-DOPA PET provides higher detection sensitivity and spatial resolution, thus allowing very thinner visual assessment of deep brain structures involvement. In this context, and except for particular follow-up study purpose [36–39], justifying semi-quantification or more sophisticated methods over visual assessment to diagnose Parkinsonism remains largely under evaluated in clinical practice. In the era of precision medicine, the search for new powerful and robust imaging biomarkers constitutes a very hot topic of interest in a wide variety of diseases. In this context, automated image processing workflows have emerged, and could facilitate the interpretation of physicians in daily practice. Motivated by its rational in high spatially resolved morphological imaging, radiomics gradually invades nuclear imaging of oncological and non-oncological diseases, including Parkinsonian syndromes [40–44]. In their very recent paper, Comte et al. trained and validated a logistic regression model with L1 regularization to identify dopaminergic denervation on ^18^F-DOPA PET/CT [44]. Among 43 first and higher-orders parameters, three textural features were found to identify abnormal ^18^F-DOPA PET almost as well as a nuclear imaging expert, considered here as the gold standard and study outcome. As mentioned by the authors, the clinical utility of such approach remains unknown, also questioning its conceptual diagnostic relevance in this particular topic, given the limited semiology of ^18^F-DOPA PET pattern abnormalities, and the well-known major logistical drawbacks of handcrafted radiomic pipelines in real-life practice. Recently, the clinical utility of deep-learning based methods to identify Parkinson disease directly from PET data has been emphasized, with very promising results [45–47]. Deep learning conceptually tackles all the limitations of handcrafted radiomics procedures and would probably constitute a more powerful and efficient alternative to human expert reading for basic imaging identification tasks. In this way, capturing the objective min SUVr from striata, which was here as relevant as visual expert assessment, appears a promising way toward simple assisted analysis workflows. However, multicentric studies are mandatory to overcome the lack of reproducibility of standard PET semi-quantitative metrics related to PET systems image reconstruction properties (the well-known center effect). In accordance with the recent EANM guidelines [11] visual analysis remains to date the most relevant parameter to predict IPD.

Our study has several limitations. Firstly, the median follow-up of 11 months could have led to potential diagnostic misclassification [48]. Because the new movement disorder society clinical diagnostic criteria [6] are currently being judged not useful by the experts in real-life practice [5] trained neurologists typically make the diagnosis on medical history, clinical symptoms (bradykinesia, rigidity, tremor) and symptoms evolution under treatment. In atypical cases, staff of experts make their conclusions on a full multidisciplinary work-up. Second, our study included outpatients with mild -early symptoms mainly, for whom clinical diagnosis was ambiguous, justifying the ^18^F-DOPA imaging (in the case of cardinal symptoms, in particular at advanced stages, dopaminergic imaging has no clinical relevance). Because our patients did not have clinical confirmation yet at the time of ^18^F-DOPA PET imaging, the severity score of IPD was not provided at this time, emphasizing the real-life practice conditions of our study. To note, this study was not designed for the PET assessment of disease clinical severity, which is out of the scope of this study and has been widely studied by the past. Third, from hundreds of quantitative morphological and metabolic measures available with Freesurfer and Petsurfer neuroimaging pipeline, only few semi-quantitative metabolic PET metrics were considered clinically relevant given the physiopathology of dopaminergic denervation of striate: SUV metabolic ratios, gradient, and asymmetry indices. This choice was motivated by the fact that (1) we wanted to compare different well-known and usable PET metrics that are easily applicable in routine practice, and more importantly transposable to the individual level which contrasts with recent radiomics studies published; (2) making statistical inference with less than 10 participants per parameter becomes conceptually unacceptable [49, 50]. Forth, the selected semi-quantitative features were competing with the median performance of five experts that blindly and independently reviewed each case to limit inter-readers potential heterogeneity. One strength of future machine learning model in this context could be the reproducibility of predictions compared to a single expert reading. The main asset in our study is the use of simultaneous PET/MRI acquisition, which improves striatal segmentation from T1-weighted sequence over PET images [51]. Nevertheless, the potential of MRI cannot be restrained to morphological analysis. Promising results showed high correlation between Parkinson disease and specific MRI-multiparametric brainstem investigation [52], in particular the iron deposit in the substantia nigra. Recent results also showed correlation between iron deposit in substantia nigra and striatal dopamine denervation seen with ^18^F-DOPA PET [53, 54]. All these results may promote further research to better capture the relevance of combining ^18^F-DOPA and MRI capabilities in this field.

## Conclusion

Visual expert analysis remains the most relevant parameter to predict IPD diagnosis at 11 months of median clinical follow-up in ^18^F-DOPA. The min SUV ratio appears interesting in the perspective of simple semi-automated diagnostic workflows.

## Data Availability

The datasets analyzed during the current study are not publicly available due relevant data protection laws but are available from the corresponding author on reasonable request.

## Abbreviations

AUC: Area under the curve
cv-ROC: Cross-validated receiver operating characteristic curves
DAT: Dopamine transporter
IPD: Idiopathic Parkinson disease
FN: False negative
FP: False positive
^18^F-DOPA: 6-Fluoro-(18F)-L-DOPA
MNI: Montreal neurological institute
MRI: Magnetic resonance imaging
PET: Positron emission tomography
SPECT: Single-photon emission computed tomography
SUV: Standard uptake value
SUVr: Standard uptake value ratio
TP: True positive
TN: True negative
